# Two different pathogenic mechanisms, dying-back axonal neuropathy and pancreatic senescence, are present in the YG8R mouse model of Friedreich’s ataxia

**DOI:** 10.1242/dmm.024273

**Published:** 2016-06-01

**Authors:** Belén Mollá, Fátima Riveiro, Arantxa Bolinches-Amorós, Diana C. Muñoz-Lasso, Francesc Palau, Pilar González-Cabo

**Affiliations:** 1 Program in Rare and Genetic Diseases and IBV/CSIC Associated Unit at CIPF, Centro de Investigación Príncipe Felipe (CIPF), Valencia 46012, Spain; 2 CIBER de Enfermedades Raras (CIBERER), Valencia 28029, Spain; 3 Cell Therapy Program, Centro de Investigación Príncipe Felipe (CIPF), Valencia 46012, Spain; 4 Department of Genetic and Molecular Medicine, Institut de Recerca Pediàtrica Hospital San Joan de Déu, Barcelona 08950, Spain; 5 Department of Pediatrics, University of Barcelona School of Medicine, Barcelona 08036, Spain; 6 Department of Physiology, Faculty of Medicine and Dentistry, University of Valencia, Valencia 46010, Spain

**Keywords:** Friedreich’s ataxia, Dying-back neuropathy, Dorsal root ganglia, Muscle spindle, Cell senescence, Islet of Langerhans

## Abstract

Frataxin (FXN) deficiency causes Friedreich’s ataxia (FRDA), a multisystem disorder with neurological and non-neurological symptoms. FRDA pathophysiology combines developmental and degenerative processes of dorsal root ganglia (DRG), sensory nerves, dorsal columns and other central nervous structures. A dying-back mechanism has been proposed to explain the peripheral neuropathy and neuropathology. In addition, affected individuals have non-neuronal symptoms such as diabetes mellitus or glucose intolerance. To go further in the understanding of the pathogenic mechanisms of neuropathy and diabetes associated with the disease, we have investigated the humanized mouse YG8R model of FRDA. By biochemical and histopathological studies, we observed abnormal changes involving muscle spindles, dorsal root axons and DRG neurons, but normal findings in the posterior columns and brain, which agree with the existence of a dying-back process similar to that described in individuals with FRDA. In YG8R mice, we observed a large number of degenerated axons surrounded by a sheath exhibiting enlarged adaxonal compartments or by a thin disrupted myelin sheath. Thus, both axonal damage and defects in Schwann cells might underlie the nerve pathology. In the pancreas, we found a high proportion of senescent islets of Langerhans in YG8R mice, which decreases the β-cell number and islet mass to pathological levels, being unable to maintain normoglycemia. As a whole, these results confirm that the lack of FXN induces different pathogenic mechanisms in the nervous system and pancreas in the mouse model of FRDA: dying back of the sensory nerves, and pancreatic senescence.

## Abstract (precis)

**Summary:** Frataxin deficiency induces different pathogenic mechanisms in the nervous system and pancreas in a YG8R mouse model of Friedreich's ataxia (FRDA). Thus, the degenerative process in FRDA is determined by the cell type.

## INTRODUCTION

The pathophysiology of Friedreich's ataxia (FRDA, OMIM 229300, ORPHA 95) affects proprioceptive neurons of dorsal root ganglia (DRG) and is associated with axonal degeneration of the posterior columns, spinocerebellar and corticospinal tracts, and a predominant involvement of large myelinated fibers of sensory nerves (Koeppen, 2011). In addition, atrophy of the dentate nucleus (DN) of the cerebellum is a major lesion in the central nervous system (Koeppen et al., 2011). In contrast to DRG, for which hypoplasia and subsequent atrophy is postulated, the DN may be normal before the onset of the disease (Koeppen and Mazurkiewicz, 2013). FRDA is a systemic disorder with heart disease as well as diabetes mellitus or glucose intolerance due to involvement of the islets of Langerhans in the pancreas. The expansion of GAA·TTC triplet-repeat sequences located in the first intron of the frataxin (*FXN*) gene is found in 98% of mutated FRDA chromosomes (Campuzano et al., 1996). The expansion of the GAA·TCC repeat elicits incorrect transcription initiation and elongation, and causes changes in chromatin, all of which are responsible for the resultant *FXN* mRNA deficiency (Kumari et al., 2011). The length of expansion is inversely correlated with the age at onset and the severity of the disorder (Monros et al., 1997).

The *FXN* gene is conserved in prokaryotes and eukaryotes (Canizares et al., 2000), which has led to the development of a large number of models in different organisms and cell lines. Owing to the characteristics of the prevalent mutation, the ideal model would be one in which FXN levels are reduced but not completely eliminated. Based on this approach, researchers have developed different disease models using either RNA interference in human neuroblastoma cells (Bolinches-Amoros et al., 2014; Palomo et al., 2011) or *Caenorhabditis elegans* (Vazquez-Manrique et al., 2006), or expressing a reduced amount of human FXN in the humanized mouse model YG8R (Al-Mahdawi et al., 2006). The YG8R mouse is a knockout mouse for the *Fxn* gene (which produces embryonic lethality) that is rescued by a transgene that contains the human *FXN* gene with a pathogenic number of repeats (90+190 GAA repeats).

In this work, we investigated the cellular effects of FXN deficiency in the YG8R mouse. Our studies confirm motor behavior abnormalities expressed as impaired motor activity, coordination and skill, and a lack of positioning for correct orientation. We found that FXN depletion causes different changes in nerve and pancreas tissue. The damage to nervous tissue begins in the axons, and neurodegeneration leads to the disappearance of neurons. In contrast, in the pancreas, senescence inhibits islet replication, with a subsequent decline in number. Low insulin production prevents normoglycemia.

## RESULTS

### Functional deficits in YG8R mice increase with age

**Fig. 1.**
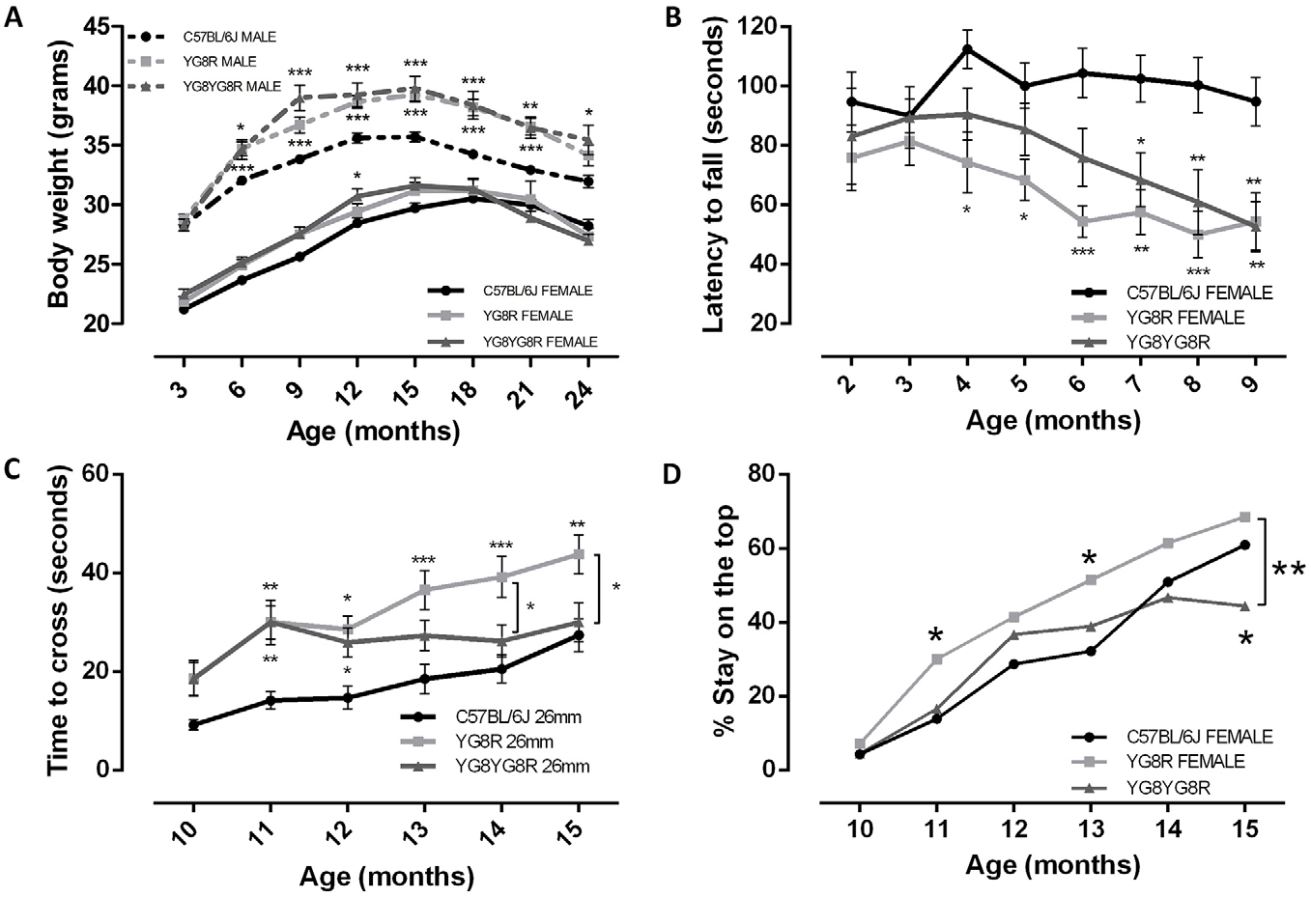
**Functional studies of YG8R and YG8YG8R (FRDA mice) and C57BL/6J mice.** (A) Weight analysis was conducted from 3 to 24 months old. Male FRDA mice showed a significant increase in weight compared with C57BL/6J, whereas female FRDA mice showed a similar tendency to WT. (B-D) Motor performance and coordination were measured in female mice using the rotarod (B), beam balance (C) and pole (D) tests. All tests confirmed a gradual effect associated with FXN expression level. (B) Rotarod analysis showed a coordination deficit of FRDA mice compared with WT. (C) Time taken to traverse the beams was measured from 10 months onwards. We show here the results obtained on a beam of 26 mm. YG8R mice took longer to traverse the beam compared with WT mice. YG8YG8R mice showed significant differences with WT in the first months but, from 13 months old, these differences disappeared. In later months the differences were obvious between YG8R and YG8YG8R. (D) The graph represents the percentage of mice that were unable to turn around in the first part of the pole test. The results indicated failure to take up the correct position for YG8R mice compared to WT. Values are expressed as mean±s.e.m.; *P≤0.05; **P≤0.01; ***P≤0.001 YG8R or YG8YG8R compared with WT. Statistically significant difference between YG8R and YG8YG8R is represented by the square brackets.

The analysis was performed in homozygous mice (YG8YG8R) containing two alleles of the transgene (equivalent to a higher level of FXN) and hemizygous mice (YG8R) containing one allele of the mutant *FXN* transgene (lower level of FXN), and C57BL/6J wild-type (WT) mice. From 6 months of age, both YG8R and YG8YG8R males showed significant weight increase compared to WT males (Fig. 1A, Table S1), as in previous studies (Anjomani Virmouni et al., 2014). Because weight is a factor that affects motor and sensory phenotyping, all performance tests were conducted in females.

The rotarod test assesses motor coordination and balance in mice. The analysis was carried out in the three genotypes of mice (Table S2) and ANOVA revealed a significant effect of both genotype (*F*=24.908, *P*<0.001) and age (*F*=2.766, *P*<0.01). Bonferroni analysis showed a significant reduction of latency time for YG8R versus WT from 4 months (*P*<0.05-*P*<0.001) and for YG8YG8R versus WT (*P*<0.05-*P*<0.01) from 7 months (Fig. 1B).

The beam balance test was performed for three beam cross-sections (26 mm, 12 mm and 5 mm) in each genotype (Table S3) and the time taken to traverse was recorded for each animal (Fig. 1C and Fig. S1A,B). ANOVA revealed a significant effect of both genotype (*F*=45.45, *P*<0.001) and age (*F*=11.688, *P*<0.001). Bonferroni analysis indicated that YG8R mice took significantly longer to traverse the beam compared with WT animals at 11 to 15 months of age (*P*<0.05-*P*<0.001) on 26- and 12-mm beams. For the 26-mm cross-section beam, the time taken by genotype YG8YG8R was significantly different from WT at 11 and 12 months of age (*P*<0.05), whereas, at 14 and 15 months of age, YG8YG8R was significantly different from YG8R (*P*<0.05). These results suggest that motor coordination and ability correlate with genotype and reduced FXN levels, which is age-dependent.

The pole test predominantly analyzes extrapyramidal motor locomotion and has previously been used to assess basal-ganglia-related movement disorders in mice (Matsuura et al., 1997; Ogawa et al., 1985). The pole test carried out on all genotypes (Table S4) found no abnormal change in the time to descend and reach the floor by any of the mouse strains (Fig. S1D); however, YG8R mice needed longer to turn and start the descent (Fig. S1C), although the differences were not significant. Likewise, the percentage of YG8R animals that did not turn around was higher compared with WT at the ages of 11 and 13 months (*P*<0.05) (Fig. 1D). These results suggest a defect in the deep senses or the position sense.

### Molecular changes determine peripheral involvement in the YG8R mouse

**Fig. 2.**
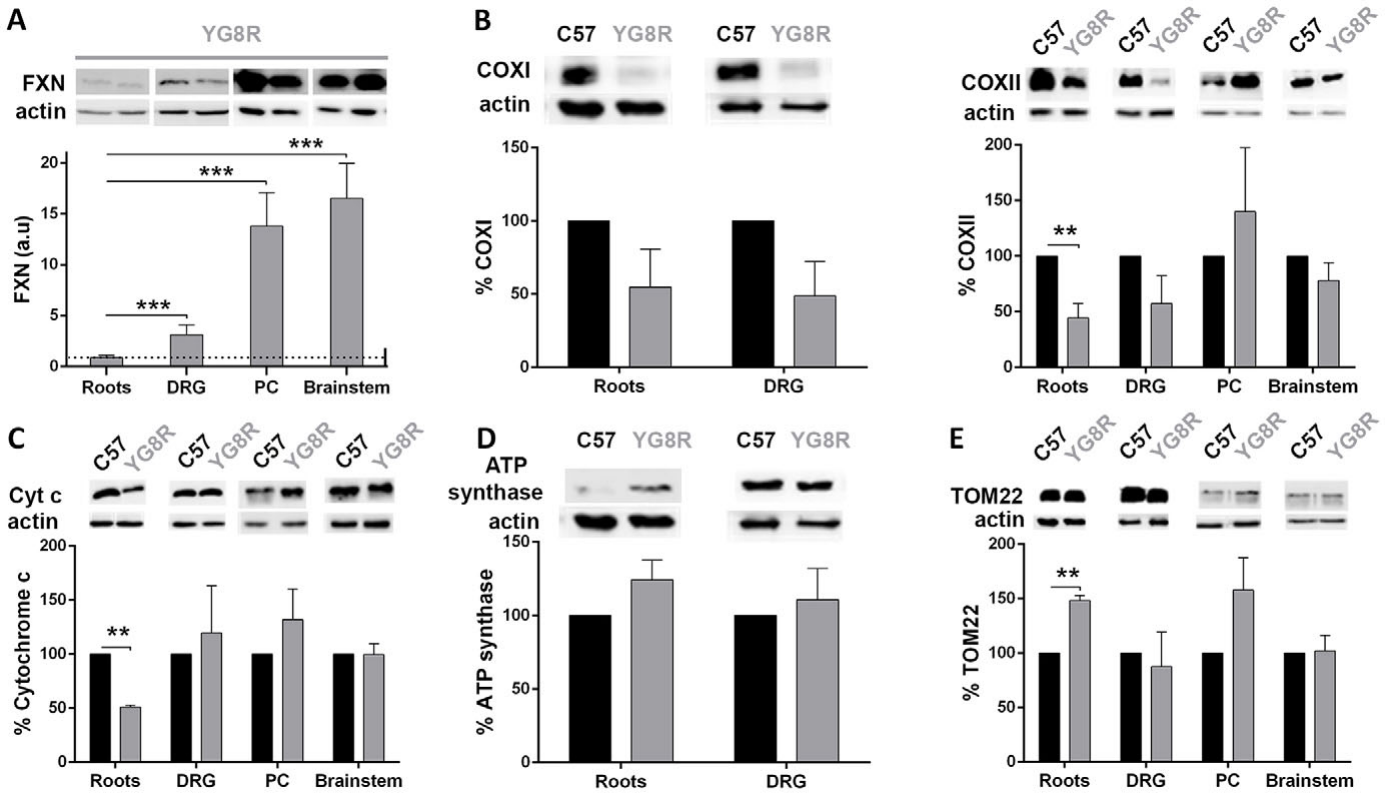
**Assessment of mitochondrial bioenergetics in neuronal tissues (brainstem, posterior columns, nerve roots and dorsal root ganglia).** A quantitative western blot assay was developed to measure FXN (A), COXI and COXII (B), cytochrome *c* (C), ATP synthase (D) and TOM22 (E) in neuronal tissues [brainstem, posterior columns (PC), nerve roots and dorsal root ganglia (DRG)]. (A) Representative western blot of human FXN expression in YG8R mice in the four neuronal tissues used in the study. Human FXN was only detected in YG8R mice, in which the transgene was present, and not in wild type (WT; data not shown). Western blot results were quantified for each lane using Fujifilm's Multi-Gauge software. To allow for loading variation, values were normalized to the actin control. Final values were expressed as a ratio to the value of FXN expression in nerve roots. DRG and nerve roots (distal tissues) expressed less FXN than the posterior columns and brainstem (proximal tissues). Values are expressed as mean±s.e.m.; ***P<0.001 neuronal tissues of YG8R (DRG/PC/brainstem) compared with YG8R roots. (B-E) Western blot results were quantified as in A, but the final values were expressed as a percentage of the C57BL/6J value. The most affected tissues were the distal tissues, especially the nerve roots. (E) Graph of TOM22 confirmed that the differences observed in the previous studies were not due to a reduction in mitochondrial number. Values are expressed as mean±s.e.m.; **P≤0.01 YG8R compared with WT (C57).

To determine the major neural structural level responsible for motor coordination dysfunction in the spinal cord, we proceeded to investigate molecular changes by western blot analysis in the YG8R mouse. We selected the DRGs, nerve roots, posterior columns of the spinal cord, and brainstem because they are affected in the neuropathology of FRDA. Studies were carried out on 19- to 22-month-old mice, the age at which motor coordination abnormalities had been observed. First, we analyzed FXN and proteins involved in biological processes related to neurodegeneration. FXN levels were reduced in nerve roots and DRGs (Fig. 2A). In contrast, levels in the brainstem were 18-fold higher than in the nerve roots. This finding suggests that a reduction of FXN levels is more evident in peripheral (nerve roots and DRG) than central (posterior columns and brainstem) neural structures.

As a major aspect of disease pathophysiology, lack of FXN induces mitochondrial dysfunction (Cnop et al., 2012; Gonzalez-Cabo and Palau, 2013; Rotig et al., 1997). Thus, we wanted to know the effects of FXN reduction on mitochondrial-associated cellular functions in the YG8R mouse. Analysis of the mitochondrial respiratory chain showed decreased levels of COXI and COXII subunits in the electron transport chain complex IV in DRG and nerve roots (Fig. 2B). Cytochrome *c*, the acceptor of electrons in complex III, was decreased only in nerve roots, whereas its levels in the other target tissues were similar to those in WT mice (Fig. 2C). In contrast, no differences in the expression of ATP synthase were observed in any tissue structure (Fig. 2D). The expression of TOM22 was also increased in the nerve roots of the YG8R mouse (Fig. 2E), which suggests that the observed changes were not due to a reduction in the number of mitochondria.

**Fig. 3.**
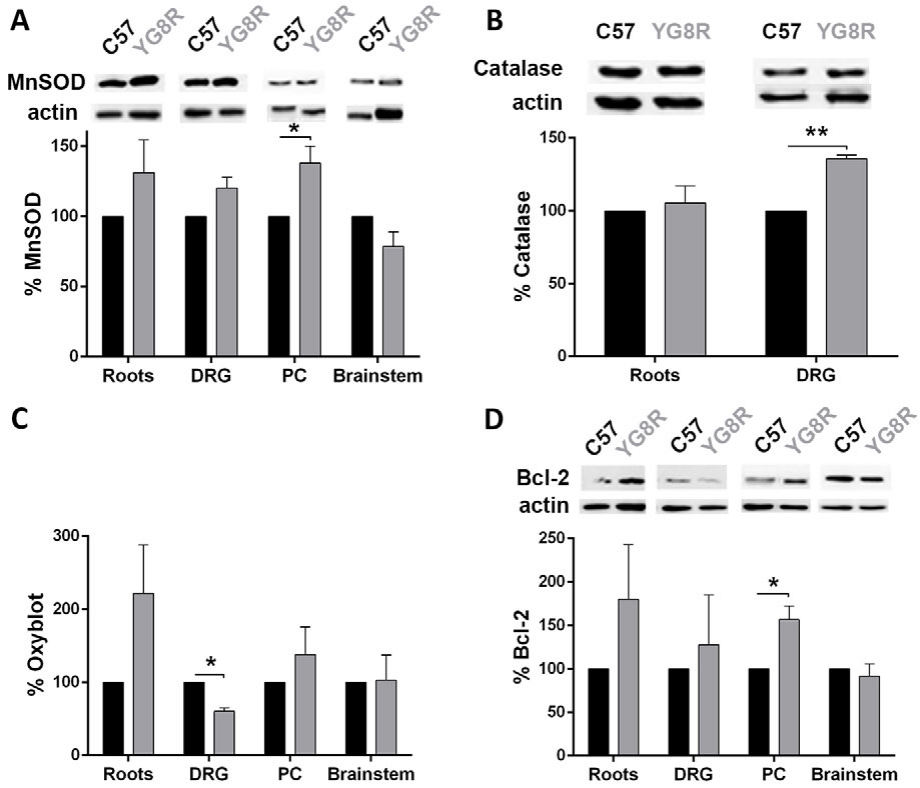
**Assessment of oxidative damage in neuronal tissues (brainstem, posterior columns, nerve roots and dorsal root ganglia).** A quantitative western blot assay was developed to measure MnSOD (A), catalase (B), carbonylated proteins (C) and Bcl-2 (D). Western blots were quantified as described in Fig. 2A, but the final values were expressed as a percentage of the C57BL/6J (WT; C57) value. The carbonylated protein results (C) showed a marked increase in nerve roots, demonstrating evidence of cellular oxidative stress, but the response of antioxidant enzymes (A,B) was limited. Elevated expression of catalase in DRG (B) could prevent oxidative stress, which was reflected in the decrease in protein carbonylation (C). Increased Bcl-2 protein (D) suggested a predisposition to survival in YG8R mice. Values are expressed as mean±s.e.m.; *P≤0.05; **P≤0.01 YG8R compared with WT. Roots, nerve roots; DRG, dorsal root ganglia; PC, posterior columns.

To gain further insight into the biology associated with mitochondria in target tissues, we measured molecular biomarkers of oxidative stress, apoptosis and autophagy. Whereas no significant changes were observed for manganese superoxide dismutase (MnSOD; Fig. 3A), catalase was significantly increased in DRG (Fig. 3B). In addition, DRG showed a significant reduction in carbonylated proteins, as suggested by oxyblot analysis, which means that the production of oxygen-derived radicals was reduced in this tissue (Fig. 3C). In contrast, although not significant, we observed a tendency for protein oxidation in nerve roots.

Other pathways reported to be altered in FXN deficiency models are apoptosis (Palomo et al., 2011) and autophagy (Bolinches-Amoros et al., 2014). In YG8R mice, we observed an increase in Bcl-2 protein in the nerve roots, DRG and posterior columns compared with WT, although the increase was only significant in the latter (Fig. 3D). Bcl-2 contributes to programmed cell death by blocking apoptotic death (Reed, 1994). We confirmed the lack of activation of caspase 3 in all tissues, and found no evidence of apoptosis (data not shown). We used LC3-II as a marker for autophagy, and the results showed no difference between YG8R and WT mice (data not shown).

Therefore, the nerve roots were the most affected tissue, followed by the DRG. These results correlated with FXN expression in the target tissue, because both tissues had lower levels of FXN than the posterior columns and brainstem. Interestingly, the altered tissues were the most peripheral, whereas the brain and spinal cord did not seem to be affected.

### Histological changes in neuronal tissues are restricted to peripheral structures such as the DRG and root ganglia

**Fig. 4.**
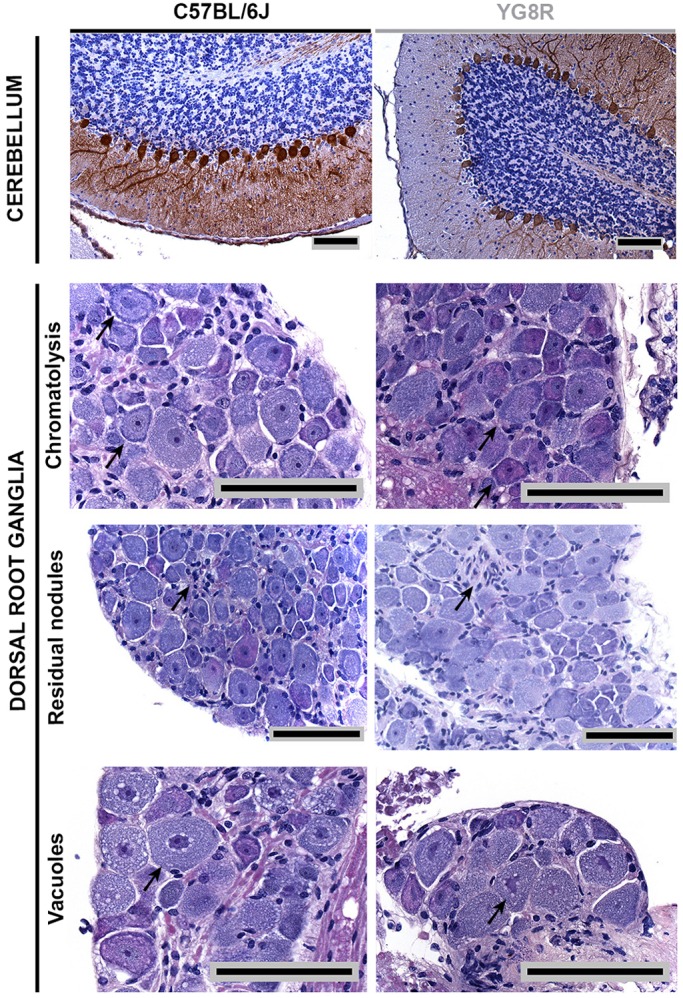
**Cerebellar cortex and dorsal root ganglia histopathology.** Immunocytochemistry with calbindin was performed in samples of cerebellum of 24-month-old YG8R and C57BL/6J mice. Purkinje cells in YG8R were similar to the control (top picture). Hematoxylin- and eosin-stained sections of lumbar DRG of 24-month-old YG8R and C57BL/6J mice were examined. We observed comparable neurodegenerative processes in both genotypes, i.e. peripheral chromatolysis, because the remains of the Nissl bodies were confined to the periphery of the neuron; satellite cells where there should have been a neuron, defined as residual nodules; and vacuoles in the cytosol. Arrows point to examples of the structures or processes that are named at the side of the figure. Scale bars: 100 μm.

**Fig. 5.**
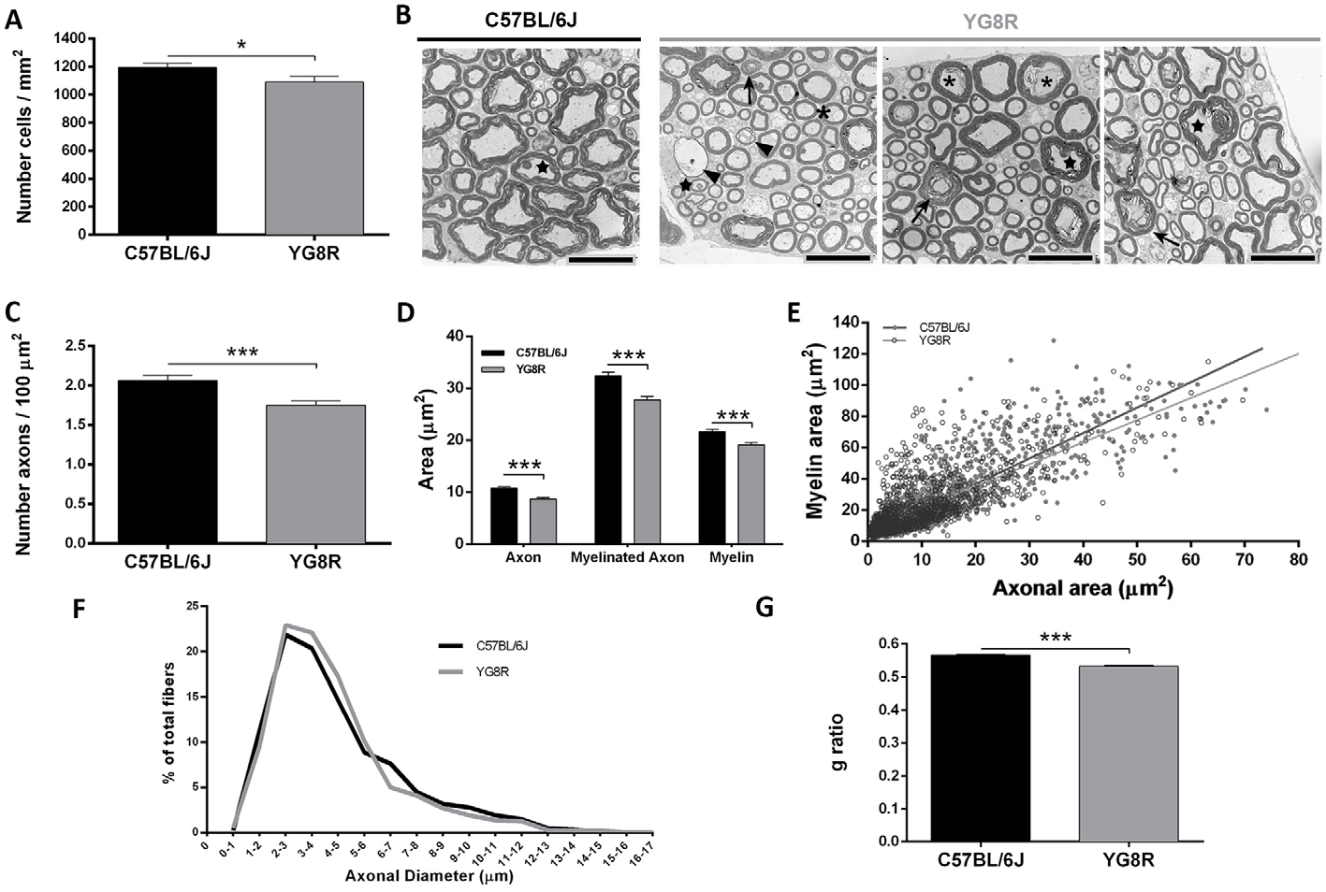
**Axonopathy in the peripheral nervous system of YG8R mice.** (A) The number of DRG neurons was significantly reduced in YG8R mice. The graph represents the number of neurons per defined area of the lumbar DRG from 24-month-old YG8R (*n*=3) and C57BL/6J (WT; *n*=3) mice. (B) Ultrastructural analysis of L5 sensory nerve rootlets from 24-month-old YG8R (*n*=3) and C57BL/6J (*n*=3) mice. Transmission electron microscopy (TEM) of the dorsal roots revealed delaminating myelin (arrow) arriving at wide and irregular shaped incisures, thinning myelin (arrowheads), infolded myelin loop (star) and enlarged adaxonal compartment (asterisk) in YG8R mice. Scale bars: 10 μm. (C) The number of axons per defined area of the dorsal roots from YG8R was significantly lower than in C57BL/6J. (D) The axon area, myelinated axon area and myelin area were measured and showed a reduction in all parameters in YG8R mice, as shown graphically. (E) Graphical representation of the relationship between axon area and myelin area. The lines are the regression lines, showing differences between the genotypes. The YG8R mouse showed a larger axon size and thinner myelin. (F) A slightly different distribution of axons was obtained by morphometric analysis of the axonal diameters from YG8R compared with C57BL/6J. (G) Quantification of the g ratio showed a significant decrease in YG8R mice compared with C57BL/6J. Values are expressed as mean±s.e.m.; *P≤0.05; ***P≤0.001 YG8R compared with WT.

Pathological changes in the DRG have previously been described in YG8R mice (Al-Mahdawi et al., 2006). The authors observed vacuoles and chromatolysis in the DRG of the lumbar region from 6-month-old animals, and lipofuscin in the DRG of 20-month-old mice. To establish the pathological changes in our model, we performed a histological study in the DRG of the lumbar region and cerebellum from YG8R and WT mice aged 6, 9, 12 and 24 months old. No changes were observed in the cerebellum: Purkinje and granule cells were normal, and no cell loss was observed (Fig. 4). In the DRG, the most obvious changes were observed at 22 months (Fig. 4), and included the presence vacuoles, chromatolysis and residual nodules (Nageotte nodules). However, all of these changes were identified in both genotypes, so can be interpreted as being associated with the aging process. However, we observed significant differences in the number of cells in the DRG, with 1226 cells/mm^2^ in the WT mice vs 1091 cells/mm^2^ in the YG8R mice (Fig. 5A). But what about the sensory axons? Did the dorsal roots show any pathological alteration besides molecular differences? To investigate further the possible relationship between axonal defects and the appearance of motor defects in older mutant animals, we performed transmission electron microscopy (TEM) analysis to obtain morphological information about the myelinated fibers of dorsal roots from 24-month-old YG8R and WT mice. Whereas most WT mice had myelinated axons with compact layers of myelin lamellae, YG8R mice had a widespread number of disrupted layers of myelin lamellae (Fig. 5B). Infolded myelin loops were present in both genotypes, but were found more frequently in YG8R axons. In YG8R mice, we observed a large number of degenerated axons surrounded by a sheath exhibiting enlarged adaxonal compartments (defined as the rim of cytoplasm of the Schwann cell and the innermost myelin layer adjacent to the axon) (Young and Boentert, 2005) or by a thin disrupted myelin sheath. We observed vacuoles and other undefined structures in the adaxonal compartment of the YG8R axons. The number of axons was significantly lower at 24 months in YG8R mice (1.75 axons/100 μm^2^) compared to WT (2.065 axons/100 μm^2^) (Fig. 5C). Morphometric analysis indicated that the myelin area of the YG8R mice was significantly lower than in WT animals (Fig. 5D), and such demyelination was more evident in the large axons (Fig. 5E). Moreover, axon area (inner area) and myelinated axon area (total area) were also reduced (Fig. 5D). In YG8R mice, we also detected changes in axonal distribution compared to WT (Fig. 5F). There were more 2- to 6-μm axons in YG8R than in WT mice. But the percentage of axons with an axonal diameter over 6 μm was lower. In addition, the g ratio (calculated by dividing the inner area by total area) was significantly reduced (Fig. 5G).

### Abnormal muscle spindle innervation in YG8R mice

**Fig. 6.**
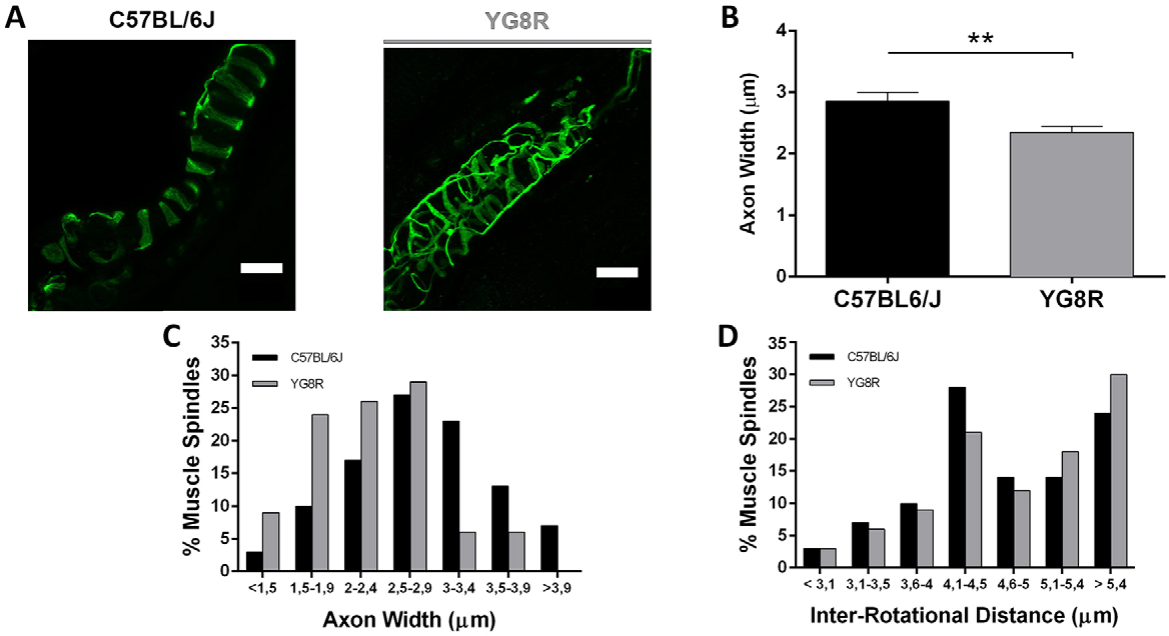
**Morphological muscle spindle Ia innervation in 24-month-old YG8R and C57BL/6J mice.** (A) Histological samples of quadriceps were examined by immunofluorescence with β-tubulin-III, which stains Ia axons. Confocal optical images from quadriceps muscle spindles showed typical annulospiral morphology in both genotypes. Scale bars: 20 μm. (B) Mean Ia axonal width was lower in YG8R than C57BL/6J (WT). (C) Axonal width distribution represented as the percentage of muscle spindles that included each size revealed an increased number of smaller Ia axons in YG8R mice. (D) Distribution of the space between the axonal rotations (IRD) represented as the percentage of muscle spindles that included each inter-rotational distance showed no differences between genotypes. Values are expressed as mean±s.e.m.; **P≤0.01 YG8R compared with WT.

Group Ia and II afferent axons of proprioceptive DRG neurons innervate muscle spindles in the periphery, providing information about balance and gait to the spinal cord. Spindle damage can alter sensorimotor function, e.g. causing incoordination. The muscle spindles from YG8R and WT mice were counted and no reduction in spindle count was detected in the YG8R quadriceps (11.5±1.7 spindles per muscle) compared with WT (13.8±2.2 spindles per muscle). Axonal width and the distance between the Ia axonal annulospiral rotations [inter-rotational distance (IRD)] were measured on a confocal microscope. These two axonal parameters were used to quantify muscle spindle group Ia innervation. Axon innervation of muscle spindles was normal in YG8R mice (Fig. 6A). In contrast, the mean axonal width of YG8R mice (2.35±0.10 μm) was significantly lower than in WT mice (2.90±0.14 μm) (*P*<0.01) (Fig. 6B), confirming the observed changes in the pattern of axonal width between the two genotypes (Fig. 6C). In addition, we observed a mild displacement in the IRD pattern of YG8R mice, with rotations that are spaced further apart (Fig. 6D).

### Loss of β-cell mass is due to senescence

*FXN* silencing in the neuroblastoma cell model induces slow cell growth associated with cellular senescence and cell cycle arrest at the G1 phase, with increased senescence-associated β-galactosidase (SA-βgal) activity (Bolinches-Amoros et al., 2014). To test whether cellular senescence occurs in neuronal tissues and contributes to the pathogenesis of the disease, we examined SA-βgal activity in the DRG and cerebellum of YG8R and WT mice aged 24 months. Histological sections of organs previously treated with X-gal solution as a substrate for β-galactosidase enzyme were examined. No differences were detected in either tissue (data not shown). We were not able to observe blue cells due to SA-βgal activity in either DNs or Purkinje cells of the cerebellum in either genotype. Regarding DRG, a similarly mild increase in SA-βgal activity was observed in YG8R and WT mice, probably associated with aging.

**Fig. 7.**
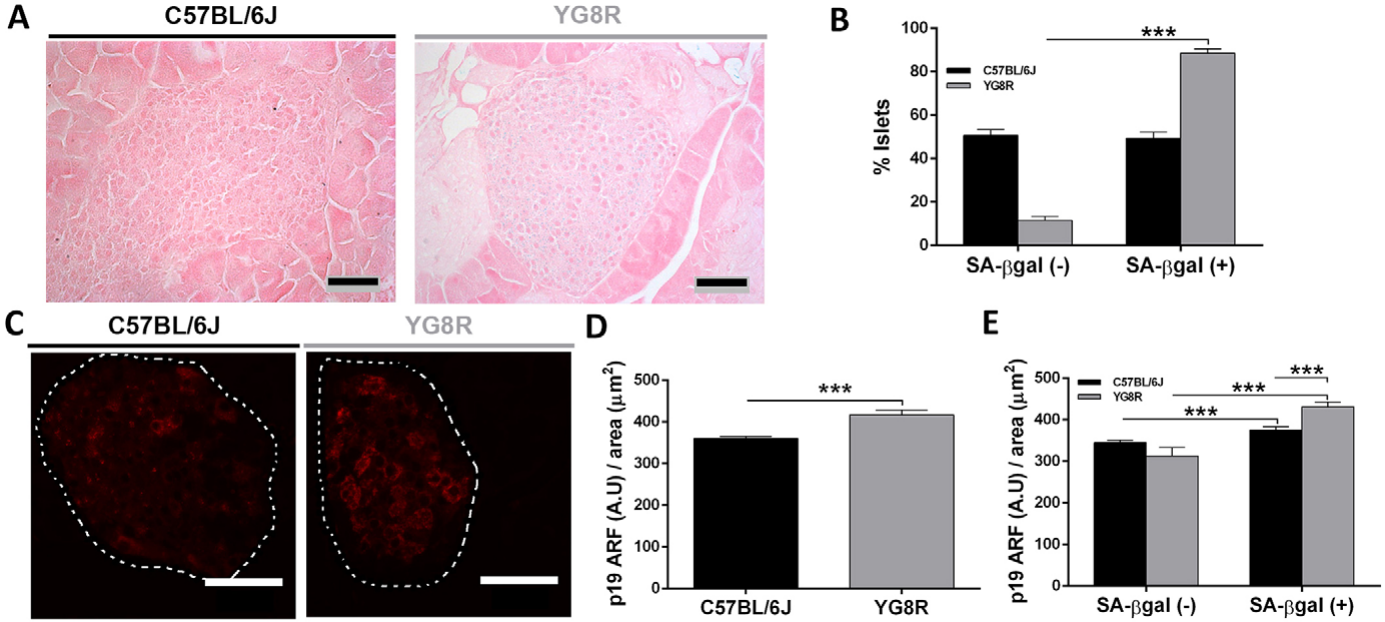
**Cellular senescence response in YG8R pancreas.** (A) Pancreas slides from 24-month-old YG8R and C57BL/6J (WT) mice previously subjected to *in situ* SA-βgal staining (blue) and eosin staining (pink) were examined by bright-field microscopy. SA-βgal staining was restricted to islets of Langerhans. (B) Evaluation of SA-βgal-positive islets showed a higher percentage of YG8R islets compared with C57BL/6J. (C) Immunofluorescence with p19 ARF antibody was performed on slides of pancreas from both phenotypes and the signal intensity per defined area showed more intense signal in YG8R mice (D). (E) The distribution of the signal intensity of p19 ARF for each SA-βgal class of islets (positive or negative) confirmed cellular senescence in the pancreas. Values are expressed as mean±s.e.m.; ***P≤0.001 YG8R compared with WT. Scale bars: 50 μm.

The other tissue affected in FRDA is the pancreas. Analysis of histological sections of pancreas treated with X-gal solution showed a significant increase in SA-βgal activity in YG8R mice. The senescent phenotype was restricted to pancreatic islets of Langerhans (Fig. 7A). Almost 90% of YG8R islets were senescent, in comparison to 50% of WT islets (Fig. 7B). Thus, aging induced the senescence phenotype but senescence was much more evident in FXN-deficient YG8R islets of Langerhans. To confirm this finding we investigated the expression of p19ARF, an additional senescence marker. Raw intensity values of p19ARF were increased in the islets of YG8R mice versus WT (Fig. 7C,D). In addition, as shown in Fig. 7E, the SA-βgal-positive islets were significantly correlated with p19ARF expression in both phenotypes, confirming the senescence phenotype.

**Fig. 8.**
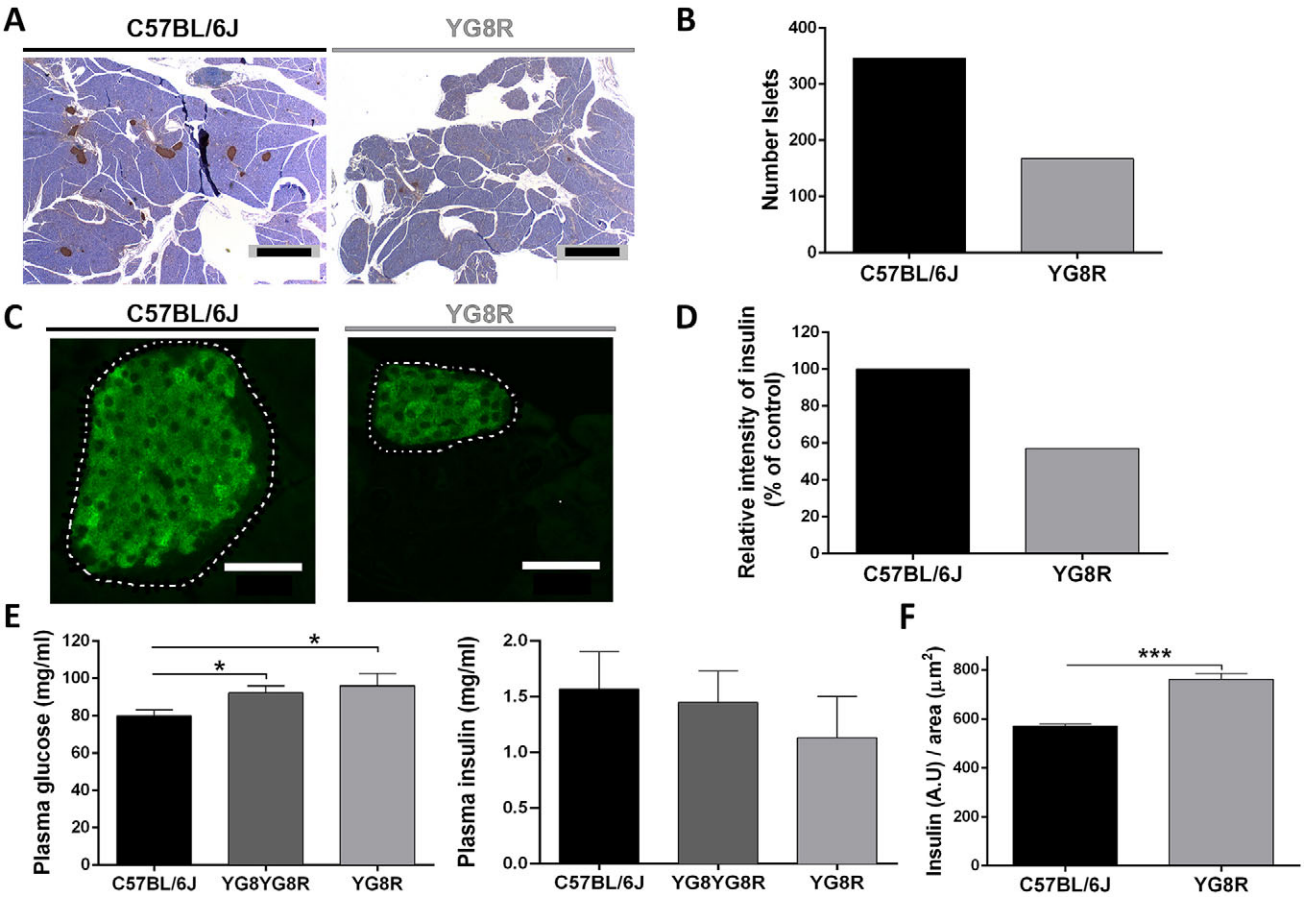
**Reduction** **of the** **number of islets and insulin secretion in YG8R mice.** (A) Representative images of immunocytochemistry with insulin on slides of C57BL/6J (WT) and YG8R mice. Scale bars: 1 mm. (B) Quantification of islets was performed on five slides per mouse from three animals of each genotype. YG8R pancreas presented fewer islets than C57BL/6J. (C) Immunofluorescence with anti-insulin antibody was performed on slides of pancreas from both phenotypes and the relative intensity signal was quantified. Scale bars: 50 μm. (D) Graph showing the lower production of insulin by YG8R pancreas. (E) Analysis of glucose and insulin in blood plasma of 20- to 22-month-old mice showed an increase in glucose due to the lower production of insulin. (F) The graph represents the insulin signal per defined area. This result revealed a normal level of insulin production by YG8R islets, because the lower β-cell mass led to the production of more insulin. Values are expressed as mean±s.e.m.; *P≤0.05; ***P≤0.001 YG8R compared with WT.

Previous results in human post-mortem pancreas of FRDA patients and mice with *Fxn* conditional knockout in pancreatic β-cells showed a decrease in islet β-cell mass (Cnop et al., 2012; Ristow et al., 2003). To address this point, we investigated pancreas islets in the YG8R mouse (Fig. 8A). The number of islets was significantly reduced in YG8R (167±9) with regard to WT mice (346±7) (Fig. 8B). We detected insulin signal (Fig. 8C) in YG8R islets; however, as the number of islets was reduced the total insulin level produced by β-cells was reduced as well (Fig. 8D). This might explain the glucose increase and insulin decrease in blood plasma of YG8R mice (Fig. 8E). However, when the insulin signal was related to β-cell area, we observed an increase in insulin content in YG8R mice (Fig. 8F), suggesting that YG8R pancreatic islets are functional and do not impair insulin synthesis.

## DISCUSSION

The YG8R mouse reproduces the molecular characteristics of the most prevalent mutation in individuals with FRDA (Al-Mahdawi et al., 2006). The transgene has a unique integration site but contains two copies of the gene. However, it was reported that there is a degree of copy-number variation in the YG8R mouse (Anjomani Virmouni et al., 2014). The comparison of FXN levels between genotypes YG8YG8R and YG8R confirmed the gradual reduction in level of the protein as the number of copies of the transgene decreased. Thus, we were able to perform some experiments in mutant animals with different FXN backgrounds.

Body-weight analysis of the mice revealed an increase in weight in YG8YG8R and YG8R males compared to WT males, as previously reported by other authors for genotype YG8R (Anjomani Virmouni et al., 2014). Thus, only females were used to investigate motor behavior. We observed significant changes between both transgenic and WT strains in the rotarod test, beam test and pole test, indicating motor incoordination in the transgenic animals. By investigating animal descent in the first part of the pole test, we found that a higher percentage of YG8R mice compared with WT were unable to turn around, indicating failure in the ability to conceive, organize and execute a sequence of actions in which proprioceptive senses are involved. After the motor-activity, coordination and ability assays, we were able to verify a pathological phenotype associated with FXN deficit. We observed more pronounced functional deficiency in YG8R mice, confirming a direct effect due to FXN deficit, and therefore the expression levels of FXN in YG8R can be considered pathological. Despite changes in the motor behavior of transgenic animals, we observed no pathological changes in the DRG or cerebellum. We reasoned that the phenotype was mild, as previously described (Al-Mahdawi et al., 2006), and we decided to perform further experiments in 24-month-old mice.

We wanted to know whether the topological level of the nervous system could be related to the pathophysiology of the disease in the YG8R model of FRDA. We investigated biochemical and morphological changes from peripheral to more central structures by analyzing the nerve roots, DRG, posterior columns and cerebellum in mice. First of all, we confirmed that there was a gradient in the level of FXN, which was lower in the dorsal roots and DRG than in central tissues, which suggests that peripheral structures might be more susceptible to the consequences of FXN deficiency. Consistent with this, changes in the mitochondrial respiratory chain were observed in nerve roots, which showed a reduction of COXII and cytochrome *c*, but this was not observed in central tissues. We did not observe significant evidence of oxidative stress, as measured by protein carbonylation. On the contrary, whereas there was a tendency to increase protein carbonylation in the nerve roots, oxidative stress was significantly decreased in the DRG. On the other hand, analysis of MnSOD and catalase suggested a tendency for an increase in oxidative stress in these tissues. As a whole, it seems that, in peripheral tissues, there is an unstable equilibrium between oxidative stress and antioxidant systems that could be related to the reduced amount of FXN. Central tissues did not show such instability. Again, when studying markers of apoptosis and autophagy, no strong evidence was observed in any tissue, suggesting that these processes are not activated in the YG8R mouse.

Morphological studies showed more relevant findings than the biochemical analysis. There was a significant reduction in the number of cells in the DRG. The analysis of distribution by axon size showed a reduction in the percentage of large fibers in YG8R mice, which might be related to the difficulties encountered by YG8R mice when performing the pole test. Histopathological study of the dorsal root revealed the important role of FXN in myelinated axons. Electron microscope images of dorsal roots showed a high number of axons that had signs of degeneration, along with a decrease in the total number of myelinated axons in YG8R mice. This result was not observed in the dorsal root of FRDA patients (Koeppen et al., 2009), in whom the large myelinated fibers had disappeared but the total number of axons was preserved owing to the excess of thinner axons. Our study only includes myelinated fibers, so the differences we observed might be due to non-myelinated fibers. We also observed that differences between axon populations regarding their size were not as evident as in patients. YG8R mice showed a reduction in myelinated fibers greater than 6 μm in diameter and increase of 2- to 5-μm fibers. Axonal atrophy is difficult to determine by loss of large fibers, but the reduced g ratio observed in the YG8R mouse supports the notion of axonal damage.

Of particular interest were the morphological observations concerning the myelinated axons in YG8R, because many had myelin-sheath decompaction and myelin invaginations. This could trigger the increase in adaxonal space observed in many fibers of YG8R mice, preventing proper connection between the Schwann cell and axon. It is well known that the increase in the size of the adaxonal compartment is the initial cause of demyelination of axons, as seen in YG8R axons. All these pathological processes could develop before axon degeneration and contribute to their disappearance. Thus, these results suggest a relevant role of the Schwann cell in the pathology of FRDA. Ultrastructural abnormalities were described in Schwann cells many years ago (McLeod, 1971) but, until the studies by Morral et al., in which sural nerve autopsies revealed clear demyelination and morphological alterations in the Schwann cell, nobody had talked about the possible role of myelin in FRDA (Morral et al., 2010). In addition, it was reported that both overexpression and reduction of FXN in glial cells in the *Drosophila* FRDA model cause degeneration in the brain (Navarro et al., 2011, 2010), confirming that FXN function is not restricted to the neurons. Moreover, the decrease in the axon area and abnormal changes in pathways involved in neurodegeneration suggest that the axon is also affected.

Few studies have been conducted in FRDA patients to determine the innervation of axons with the specialized structures localized in skin and muscle. Nolano and collaborators showed impoverished cutaneous innervation in FRDA skin biopsies (Nolano et al., 2001). Their results on skin biopsies differed from previous results on sural nerves in which no loss of unmyelinated fibers was observed (McLeod, 1971; Morral et al., 2010). In our case, in order to understand the defects that we had observed in sensorimotor behaviors such as balance, proprioception regulation and coordination in YG8R mice, we studied the histology of the muscle spindles and innervation of the sensory afferent fiber group Ia. The number and morphology of the muscle spindles was normal, as was the innervation of the neuron. However, the axonal width had a smaller range than the WT, according to results obtained from the dorsal root. Multiple studies in humans (Macefield et al., 2011) and in mouse models of neuropathies (Muller et al., 2008) suggest that morphological changes or loss of functionality in the muscle spindles could be the cause of ataxic gait. In summary, we concluded that the distal nerve structures of YG8R mice (muscle spindle, DRG and dorsal root) were more affected than other tissues and also were the origin of the pathology. In old age, this pathology would advance to the central nervous system, consistent with the dying-back process described in patients.

Senescence occurs naturally in aging-related degeneration, although recently reported findings confer on senescence a relevant role in neurodegenerative diseases, contributing to the neuronal injury observed in these disorders. We have previously demonstrated the presence of senescence-associated β-galactosidase activity in a FXN deficiency model in SH-SY5Y neuroblastoma cells, and we hypothesized that senescence could be important for neuron development, which could explain the hypoplasic changes observed in the spinal cord of postmortem studies from FRDA patients (Bolinches-Amoros et al., 2014). Thus, we checked senescence in the YG8R mouse. We found no evidence of senescence in the DRG and cerebellum, but observed it in the pancreas, more specifically, in the islets of Langerhans. In fact, up to 90% of YG8R islets but only 50% in the WT were positive for senescence markers, which suggests that this is the effect of FXN depletion. As observed in other mouse models and in human studies, we found that the number of islets and the total amount of insulin produced by the islets decreased. This finding was also observed in the blood plasma associated with increased blood glucose levels. Hyperglycemia and reduction of plasma insulin have been attributed to abnormal islet function. However, on the contrary, we observed that senescent islets were able to produce insulin and in greater quantities relative to area. Similar findings have been observed in the pancreas of FRDA patients, in which the insulin production area was smaller, but the intensity of staining of islet insulin was similar to that in controls (Cnop et al., 2012). We believe that this increase in the production of insulin is due to efforts made by the cell to maintain normal levels of blood glucose. Ultimately, however, production levels are insufficient to meet physiological needs. Diabetes in FRDA has been attributed to the induction of apoptosis and a decrease in the proliferation of β-cells (Cnop et al., 2012; Igoillo-Esteve et al., 2015; Ristow et al., 2003). We found that β-cells are not able to divide because they enter the senescence pathway and that the number of cells lost is more than that caused by apoptosis. Regulation of the number of β-cells is dependent on replication rather than on differentiation from stem cells, this being the main mechanism of regulation of insulin production (Tavana and Zhu, 2011). The high proportion of senescent islets in YG8R mice makes the β-cell number and islet mass decrease to pathological levels, because they are unable to maintain normoglycemia. If prolonged in time, this situation would lead to the onset of diabetes that affects some patients. Therefore, this mouse model is interesting for studying the early stages of diabetes in patients with Friedreich's ataxia. And later, in advanced phases, both oxidative stress (Igoillo-Esteve et al., 2015; Ristow et al., 2003) and reticulum stress (Cnop et al., 2012) will trigger apoptotic cells, reducing islet mass.

Generating a good mouse model that reproduces the pathology of individuals with FRDA remains a challenge. The YG8R mouse shows a mild phenotype that is evident at advanced ages. Here, we have characterized the histopathology of the YG8R mouse in more detail, including new structures previously not investigated (nerve roots, muscle spindle and pancreas) and others previously studied (DRG and cerebellum). We have confirmed that there is a loss of myelin in the axons of the neurons from the DRG, possibly owing to disruption of the adaxonal myelin and the loss of connection between Schwann cells and axons. This phenomenon together with axonal shrinkage due to neurodegenerative processes suggests that the pathophysiological process is caused both by defects in the axon and Schwann cell. Most striking is that the pancreatic response is different from that of neuronal tissues. We propose that the senescence observed in the islets of Langerhans is a trigger of the pathophysiological process observed in the pancreas.

## MATERIALS AND METHODS

### Animals

YG8R mice were purchased from The Jackson Laboratory Repository (Stock no. 008398). Animals were group-housed under standard housing conditions with a 12 h light-dark cycle, and food and water *ad libitum*. Mice used in this study originated from a colony of YG8R×YG8R. Transgene copy number was verified for every animal using quantitative real-time PCR (qPCR). We used both homozygous mice, containing two alleles of the transgene (referred to as YG8YG8R; equivalent to higher levels of FXN protein), and hemizygous mice, containing one allele of the mutant *FXN* transgene (referred to as YG8R; with the lowest level of FXN), and C57BL/6J wild-type (WT) mice. The method for euthanasia was cervical dislocation. All mouse experiments were approved by the local Animal Ethics Review Committee of Consejo Superior de Investigaciones Científicas (CSIC) and Centro de Investigación Príncipe Felipe (CIPF).

### Behavioral testing

Weights and survival points from the WT, YG8R and YG8YG8R animals were measured monthly. The cohort of female mice was used to measure motor activity (WT *n*=25; YG8YG8R *n*=18; YG8R *n*=14).

#### Rotarod

Female mice were tested monthly, starting at 2 months and ending at 9 months of age. The mice were trained for 4 consecutive days before the first test was performed. On the first 2 days, a training trial of 1 min at 4 rpm on the rotarod apparatus was included followed by 1 min with a progressive increase in speed from 0 to 40 rpm during the last 2 days. Each animal was tested over 4 consecutive days and each daily session included four trials (with an inter-trial interval of 10 min) during which the speed of the rod changed from 0 to 40 rpm over 300 s. Latency to fall was recorded in seconds.

#### Balance beam

Sensory-motor coordination was tested using balance beams (100 cm length; 26, 12 and 5 mm cross-section). The balance beam was elevated 50 cm above the floor. Female mice were tested monthly, starting at 10 months and ending at 15 months of age. Mice were trained 1 week before the first test was done and each mouse had to walk across the beam (26, 12 and 5 mm) three times. Each mouse performed three trials per beam with an inter-trial interval of 10 min. Trials in which the animal took longer than 60 s to cross or fell off the beam were not scored.

#### Pole test

Mice were placed head upward at the top of a vertical pole (55 cm high with rough surface and 8 mm diameter). Female mice were tested monthly, starting at 10 months and ending at 15 months of age. The mice were trained 1 week before the first test and each mouse had to turn around and descend five times consecutively. The pole test consisted of five trials. The time taken to turn was measured first. The time taken to descend the vertical rod was recorded. A maximum time of 120 s was allowed for executing the task.

### Histological grading

#### Cerebellum

YG8R and C57BL6/J mice were killed at 6, 9, 12 (*n*=1) and 24 (*n*=3) months. Each cerebellum was fixed in 4% PFA in 1× PBS for 24 h at 4°C. Sagittal sections were prepared from paraffin-embedded tissue blocks and slides were stained with hematoxylin and eosin. Immunohistochemistry assays were performed after dewaxing paraffin sections with calbindin D28K antibody (Sigma-Aldrich) and biotinylated donkey anti-mouse F(ab)_2_ was used as the secondary antibody.

#### Dorsal root ganglia (DRG)

YG8R and C57BL6/J mice were killed at 6, 9, 12 (*n*=1) and 24 (*n*=4 C57BL6/J and *n*=3 YG8R) months. L4 and L5 DRG were fixed in 4% PFA in 1× PBS for 30 min at room temperature. DRG were sectioned from paraffin-embedded tissue blocks and slides were stained with hematoxylin and eosin.

#### Dorsal nerve root

YG8R and C57BL6/J mice were killed at 24 months (*n*=3). Transmission electron microscopy (TEM) tissue preparation was conducted as described previously (Arnaud et al., 2009) with some modifications. Vertebral columns were dissected and post-fixed by immersion in 2% PFA and 2.5% glyceraldehyde in 1× PBS and shaken overnight at 4°C. The following day, dorsal roots were dissected and washed in 0.1 M cacodylate buffer overnight. On the third day samples were osmicated for 1 h in 1% OsO_4_ in cacodylate buffer at 4°C. Dorsal roots were washed in water, dehydrated and embedded in propylenoxide/epoxy resin and araldite (Durcopan). Ultrathin sections (0.8 μm) were cut and stained with 2% uranyl acetate.

#### Muscle spindle

YG8R and C57BL6/J mice were killed at 24 months (*n*=3). The quadriceps femoralis were dissected and fixed in 4% PFA in 1× PBS for 6 h at 4°C. The cryoprotection protocol consisted of a saccharose gradient (10%, 20% and 30%) performed before freezing. The samples were placed in OCT embedding medium (Thermo Scientific) and frozen, sectioned in 50-μm longitudinal serial sections and mounted on Superfrost slides. Sensory axons were visualized using anti-β-tubulin-III (Sigma-Aldrich). One muscle per genotype (YG8R and C57BL6/J) was analyzed and all muscle spindles of each muscle were imaged for spindle innervation quantification, which was performed as described elsewhere (Muller et al., 2008).

#### Islets of Langerhans

YG8R and C57BL6/J mice were killed at 24 months (*n*=3). Each pancreas was sectioned from paraffin-embedded tissue blocks and slides were stained with hematoxylin and eosin.

### Analysis of SA-βgal activity

Tissues (DRG, cerebellum and pancreas) were fixed in 4% formaldehyde for 2 h, washed with PBS and stained with staining solution for 7 h {40 mM citric acid/Na phosphate buffer, 5 mM K_4_[Fe(CN)_6_] 3H_2_O, 5 mM K_3_[Fe(CN)_6_], 150 mM sodium chloride, 2 mM magnesium chloride and 1 mg per ml X-gal in distilled water}. After washing with PBS, tissues were post-fixed in 4% formaldehyde overnight and embedded in paraffin. Slides were counterstained with eosin.

### Western blot

DRGs, nerve roots, posterior columns of spinal cord and brainstem were collected (WT *n*=4; YG8R *n*=4) from mice at 22-24 months of age, frozen on dry ice and stored at −80°C until further processing. For western blot analysis, tissues were mechanically homogenized in 300-500 μl homogenizing buffer [Tris-HCl pH 7.4 50 mM, Triton X-100 1%, MgCl 1.5 mM, NaF 50 mM, ethylenediaminetetraacetic acid (EDTA) 5 mM, sodium orthovanadate 1 mM, phenylmethylsulfonyl fluoride (PMSF) 0.1 mM, dithiothreitol (DTT) 1 mM and protease inhibitor (Roche) 1×]. Only DRGs and nerve roots were ultrasonicated at 10 Amp for 15 s. All homogenates were centrifuged at 13,000 ***g*** for 10 min at 4°C and the supernatant collected. Protein extracts were resolved by SDS-PAGE and transferred to polyvinylidene difluoride (PVDF) membrane. Membranes were stained with specific antibodies: anti-FXN (MAB-10485, Immunological Sciences), anti-COXI (MS404, Mitosciences), anti-COXII (A6404) and anti-ATPase 5-subunit α (459240) (Molecular Probes), anti-cytochrome *c* (556433, BD Biosciences), anti-TOM22 (HPA003037, Sigma), anti-SOD2 (MAB0689, Abnova), anti-catalase (C0979, Sigma), anti-BCL2 (2870) and anti-caspase-3 (9661) (Cell Signaling). Equal protein loading was assessed using an antibody against actin (Sigma). After incubation with the appropriate secondary antibodies, protein bands were detected using a Fujifilm Las-3000 after incubation with the ECL Plus Western Blotting Detection System (GE Healthcare). The density of the bands was quantified using Multi Gauge V2.1 software.

### Oxidative stress assays

Protein carbonylation analysis was performed as previously described (Bolinches-Amoros et al., 2014).

### Morphometric analysis

#### Dorsal nerve root

At least ten 2550×-magnification non-overlapping TEM (FEI Tecnai G2 Spirit; FEI Europe) images of each dorsal root were digitalized using a Morada digital camera (Olympus Soft Image Solutions GmbH). Morphometric analysis was performed using the plug-in g-ratio calculator (developed at the University of Lausanne; http://cifweb.unil.ch) of ImageJ. Myelinated axons were counted manually. The number of axons analyzed was C57BL6/J *n*=3, 1997 axons and YG8R *n*=3, 1653 axons. The inner limit of the myelin sheath was defined as the axonal area. The outer limit of the myelin sheath was defined as the myelinated axon area. The myelinated axon area minus the axon area was defined as the myelin area.

#### Islets of Langerhans

YG8R and C57BL6/J mice were killed at 24 (*n*=3) months. Islet morphometric analysis was performed on five non-consecutive longitudinal sections of the pancreas per animal. Slides were digitized using a Hamamatsu camera (Tokyo, Japan) connected to a Leica DMR microscope (Nussloch, Germany). All images were captured under constant exposure time, gain and offset. To quantify insulin signal and p19 ARF signal, we collected fluorescence images of all islets present on the slides for each genotype. We then measured the pixels produced by fluorescence using ImageJ and determined the fluorescence level relative to the islet area minus the 4′,6-diamidino-2-phenylindole (DAPI) area.
